# Vasoactive intestinal peptide axis is dysfunctional in patients with Graves’ disease

**DOI:** 10.1038/s41598-020-70138-3

**Published:** 2020-08-03

**Authors:** M. Carrión, A. M. Ramos-Leví, I. V. Seoane, R. Martínez-Hernández, A. Serrano-Somavilla, D. Castro, Y. Juarranz, I. González-Álvaro, Rosa P. Gomariz, Mónica Marazuela

**Affiliations:** 1 Departamento de Biología Celular, Facultad de Biología, Universidad Complutense de Madrid, Calle José Antonio Novais, 12, 28040 Madrid, Spain; 2 Servicio de Endocrinología Hospital Universitario de la Princesa, Instituto de Investigación Sanitaria la Princesa, 28006 Madrid, Spain; 3 Servicio de Reumatología Hospital Universitario de la Princesa, Instituto de Investigación Sanitaria la Princesa, 28006 Madrid, Spain

**Keywords:** Immunology, Endocrinology

## Abstract

Vasoactive intestinal peptide (VIP) is a neuropeptide with potent immunoregulatory properties. Reduced serum VIP levels and alterations in VIP receptors/signaling on immune cells have been associated with different inflammatory/autoimmune diseases. However, its role in autoimmune thyroid diseases (AITD) remains unknown. This study examined the interrelationship between VIP system, autoimmune background and thyroid hormones in peripheral immune cells in patients with AITD. Only Graves’ disease (GD) patients showed significantly lower serum VIP levels when compared to healthy subjects and to Hashimoto’s thyroiditis patients. Serum VIP levels were lower at the onset of GD, showing a significant negative correlation with thyroid hormone levels. The expression of VIP receptors, VPAC1 and VPAC2, was significantly upregulated in peripheral blood mononuclear cells (PBMC) from GD patients. There was an impairment of VIP signalling in these patients, probably attributable to a dysfunction of VPAC1 with preservation of VPAC2. The correlation between VPAC1 and thyroid hormone receptor expression in PBMC from healthy subjects was lost in GD patients. In summary, the VIP system is altered in peripheral immune cells of GD patients and this finding is associated with different thyroid hormone receptor patterns, showing a dynamic inter-regulation and a prominent role of VIP in this setting.

## Introduction

Experimental data and clinical observations in animal models and humans point to an important role of interactions between neuroendocrine and immune systems for the maintenance of the overall homeostasis^1,2^. This bidirectional network is functionally supported by the presence of shared signalling molecules, including hormones, neuropeptides, cytokines, and their respective receptors. Thus, neuropeptides and hormones are able to modulate immune functions, and immune mediators are capable of affecting the endocrine system. Therefore, although the immune system itself has mechanisms of self-regulation, the neuroendocrine system is also involved in its control. Accordingly, impairment of mechanisms mediating such regulatory relationships has been linked with the development of autoimmune diseases^3^.

Thyroid hormones, triiodothyronine (T3) and thyroxine (T4), are implicated in key physiological processes, including development, differentiation, and regulation of metabolism^4^. Besides, accumulating evidence proves the impact of thyroid status on immune responses and inflammation, by directly affecting the functional activity of monocytes, macrophages, lymphocytes and natural killer^3^. Biological actions of thyroid hormones are mediated by classical signalling mechanisms initiated through binding to their corresponding nuclear receptors, TRα and TRβ, that directly modulate gene transcription^5^. In addition, recent investigations have described that they also act at the plasma membrane via integrin αvβ3, triggering signalling cascades that indirectly contribute to the regulation of gene expression^4^.

Thyroid autoimmune diseases (AITD) include a broad spectrum of disorders, with Graves’ disease (GD) and Hashimoto’s thyroiditis (HT) being the most frequently observed^6–8^. GD is characterized by the presence of serum autoantibodies directed against the thyrotropin (TSH) receptor (TRAb), which over-activate this receptor in thyrocytes, leading to thyroid hormone hyperproduction and unrestrained release. At the time of diagnosis, GD patients exhibit serum FT4 levels above the normal reference range and are classified as hyperthyroid patients. There are different treatment options available for GD such as antithyroid drugs, radioiodine and surgery (thyroidectomy), which may allow some patients to recover the normal FT4 levels, thus being classified as euthyroid GD patients. On the other hand, HT represents the archetype for T-cell-mediated degenerative diseases. It is characterized by a progressive autoimmune thyrocyte depletion, resulting in impaired thyroid hormone production and clinical hypothyroidism^9^.

Vasoactive intestinal peptide (VIP) is a homeostatic peptide secreted by nerve endings, endocrine and immune cells, with potent immunoregulatory and anti-inflammatory properties. The main signalling pathway mediating VIP effects is activation of adenylate cyclase (AC) through its specific Protein G coupled receptors, VPAC1 and VPAC2^10^. The role of VIP in inflammatory disorders has been broadly reported. Specifically, exogenous VIP administration exhibits beneficial effects in murine models of inflammatory/autoimmune disorders, by reducing immune reactions and inducing anti-inflammatory mediators^11,12^. Moreover, in vitro studies on human cells have validated the ability of VIP to reshape both innate and adaptive immune responses. VIP impairs acquisition of the macrophage proinflammatory polarization profile^13^^,^ and alters the Th1/Th2 balance in CD4 T cell differentiation in favour of Th2 cells, stimulating the acquisition of a Th17 non-pathogenic profile and inducing regulatory T cells (Treg)^14^. Furthermore, reduced serum VIP levels have been described in different inflammatory/autoimmune diseases, emerging as a potential prognostic biomarker in patients with early arthritis^15,16^ and early spondyloarthritis (SpA)^17^. In this sense, alterations in the expression and functionality of VIP receptors have also been observed in these autoimmune disorders, portraying an association with disease activity^18,19^.

Given the relationship between thyroid and immune function, our hypothesis was that both the autoimmune process and the alterations of thyroid hormone levels in AITD could be interrelated with changes in the VIP system, which, in turn, would modulate the immune response. Therefore, we first explored if serum VIP levels were altered in patients with GD or HT, and if these alterations were related to relevant clinical parameters or associated with different thyroid status. We then studied the expression and function of VIP receptors in peripheral blood mononuclear cells (PBMC). In addition, we characterized the expression pattern of thyroid hormone receptors in order to elucidate if alterations in either of these systems in PBMC could be involved in thyroid autoimmune diseases.

## Results

### Serum VIP levels are decreased in hyperthyroid GD patients

Given that a decreased expression of VIP has been reported in several autoimmune and inflammatory diseases, we first evaluated serum levels of VIP in GD and HT patients, in an attempt to explore its relevance in two clinically opposed thyroid autoimmune diseases. We also evaluated thyroid hormone levels and thyroid autoantibodies (Table 1a).

**Table 1:** Demographic and clinical characteristics of AITD patients and healthy donors. (**a**) Patients with different autoimmune thyroid diseases and healthy donors. (**b**) Subgroups of patients with Graves’ disease.

a	HD (n = 49)	HT (n = 78)	GD (n = 144)	*P *value
Age (years)	NA	49 (35–55)	49 (37–56)	0.51
Sex (female)	NA	63	114	0.777
FT4 (ng/dl)	NA	1.17 (0.93–1.40)	1.60 (1.14–2.55)	0.000
TSH (uU/ ml)	NA	4.01 (2.09–6.67)	0.01 (0.00–0.98)	0.000
TPOAb(UI/ml)	20 (0–20)	438 (174–771)	155 (20–461)	0.000
TgAb (UI/ml)	20 (0–20)	336 (20–991)	20 (20–557)	0.002
TRAb (U/l)	0 (0–0)	0.47 (0.19–0.69)	2.70 (1.28–7.63)	0.000
VIP (pg/ml)	364.11 (331.11–416.88)	361.42 (309.28–424.03)	334.24 (303.75–373.58)	0.003

b	Euthyroid GD (n = 36)	Hyperthyroid GD (n = 73)	Hypothyroid GD (n = 35)	*P *value
Sex (female)	30	55	29	0.518
Age (years)	48 (40–54)	47 (33–57)	52 (33–60)	0.773
FT4 (ng/dl)	1.12 (0.93–1.27)	2.42 (1.95–3.36)	1.19 (0.72–1.57)	0.000
TSH (uU/ ml)	0.24 (0.01–1.94)	0 (0–0.01)	2.31 (0.5–10.16)	0.000
TPOAb (UI/ml)	155 (20–459)	165 (20–613)	70 (20–300)	0.297
TgAb (UI/ml)	20 (20–319)	20 (20–649)	20 (20–552)	0.156
TRAb (U/ml)	2.39 (1.2–9.89)	2.72 (1.58–8.0)	3.21 (0.83–7.21)	0.015
VIP (pg/ml)	346.07 (318.42–394.23)	325.08 (295.73–351.29)	353.63 (325.79–389.92)	0.000

Values are given as the median and (interquartile range). HD (healthy donor), HT (Hashimoto’s thyroiditis), GD (Graves’ disease), FT4 (free thyroxine 4), TSH (thyroid-stimulating hormone), TPOAb (thyroid peroxidase antibody), TgAb (thyroglobulin antibody), TRAb (TSH-receptor antibody), VIP (vasoactive intestinal peptide), NA (not available). Normal reference values for hormones and autoantibodies are: FT4 (ng/dl): 0.93–1.7; TSH (uU/ml): 0.27–5; TPOAb (UI/ml) < 100; TgAb (UI/ml) < 344; TRAb (U/l) < 0.7. *P* values are shown for Chi-square test (categorical values) and Kruskal–Wallis test (continuous variables). For more details, see “Methods” section.

**Figure 1:**
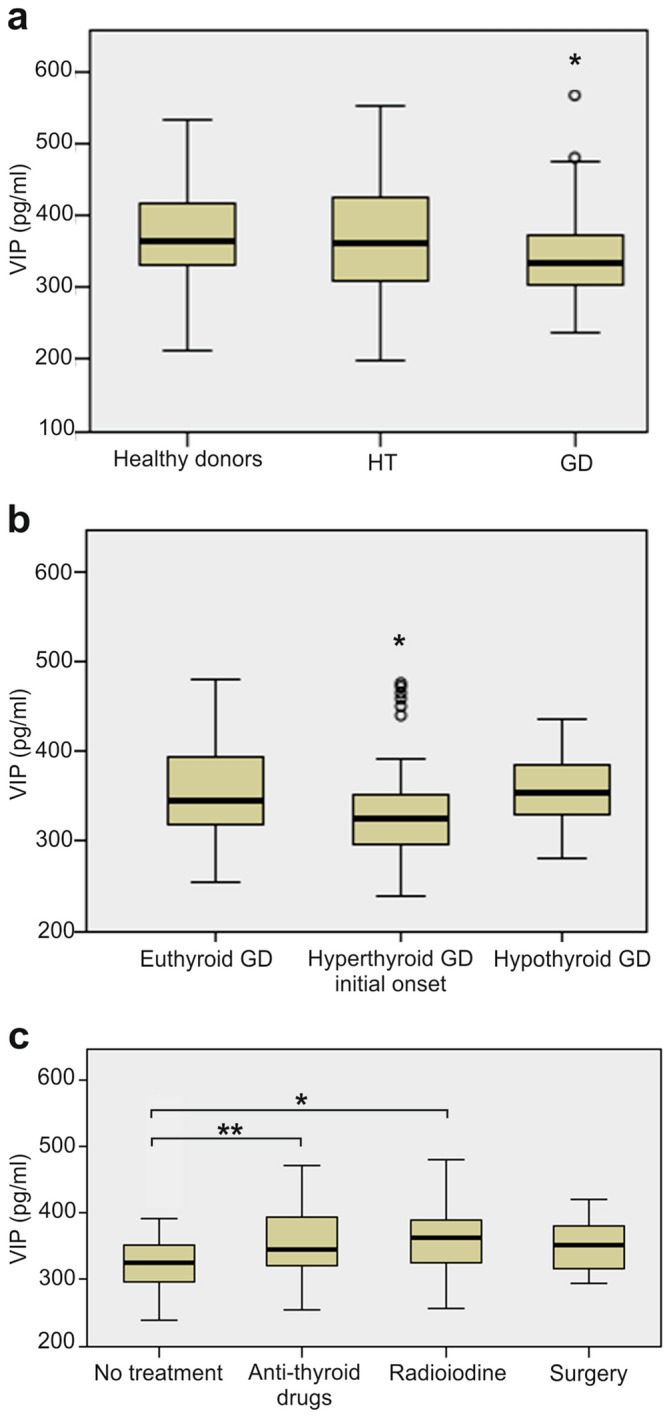
VIP serum levels in patients with different thyroid function status. (**a**) Association between VIP levels and thyroid status. ELISA determinations of VIP serum levels in 78 patients with Hashimoto’s thyroiditis (HT), 144 with Graves’ disease (GD), and 49 healthy donors (HD). (**b**) Association between VIP serum levels and thyroid function in patients with GD. ELISA determinations of VIP concentration in serum from 73 GD patients at the initial onset of the disease, 36 euthyroid GD patients controlled with antithyroid drugs and 35 GD hypothyroid patients due to treatment iatrogenesis. (**c**) Association between VIP levels and treatment groups in patients with GD. ELISA determinations of VIP concentration in serum from 73 GD patients with no treatment, 36 treated with anti-thyroid drugs, 21 treated with radioiodine and 14 that underwent surgery. Data are presented as the interquartile range (p75 upper edge of the box, p25 lower edge, p50 midline), p90 (line above the box), and p10 (line below the box) of serum VIP levels. Dots represent outliers. *P < 0.05; **P < 0.01.

Regarding serum VIP levels, there were no differences between sexes and no significant correlation with age were observed. Likewise, no differences were detected between smokers and non-smokers, or in patients with or without family past medical history of autoimmune or thyroid disease. Interestingly, only GD patients showed significantly lower serum levels of VIP (median normalized: 334.24 pmol/ml) when compared to healthy subjects (364.11 pmol/ml) and with HT patients (361.42 pmol/ml) (Fig. 1a). Correlation and regression analysis were performed between VIP serum levels and relevant clinical parameters on each group of patients, including serum levels of FT4, TSH, TPOAb, TgAb and TRAb, age and sex. These analyses only revealed a significant negative correlation between VIP serum levels and FT4 levels in GD patients (B = − 7.709, *P* = 0.021), but no other significant relationships were found (data not shown).

To further examine the altered levels of VIP found in the group of GD patients, VIP serum concentrations were analysed in relation to the thyroid hormone status of these patients. VIP levels were only decreased in recently diagnosed hyperthyroid GD patients but not in euthyroid or hypothyroid patients after therapy (Table 1b, Fig. 1b). Specifically, patients who had not received any prior treatment for hyperthyroidism had the lowest median serum VIP levels (325.08 pg/ml) compared to patients who had received anti-thyroid drugs (345.01 pg/ml, *P* = 0.006) or radioiodine (361.92 pg/ml, *P* = 0.024). The number of patients that had undergone previous surgery was lower, but we also observed a trend for higher VIP levels (350.65 pg/ml) than treatment-naïve patients (*P* = 0.065). Interestingly, we did not find significant differences in serum VIP levels between the three treatment groups (Fig. 1c). Therefore, our results revealed that diminished VIP levels in GD patients are only associated with the hyperthyroid status.

### Increased expression and altered function of VIP receptors, VPAC1 and VPAC2, in PBMC from GD patients

Once confirmed that serum VIP levels were only reduced in hyperthyroid GD patients and given that we hypothesized that the alterations of thyroid hormone system might be interrelated with the VIP axis, we next examined the expression and functionality of VIP receptors in PBMC from both euthyroid and hyperthyroid GD patients.

**Figure 2:**
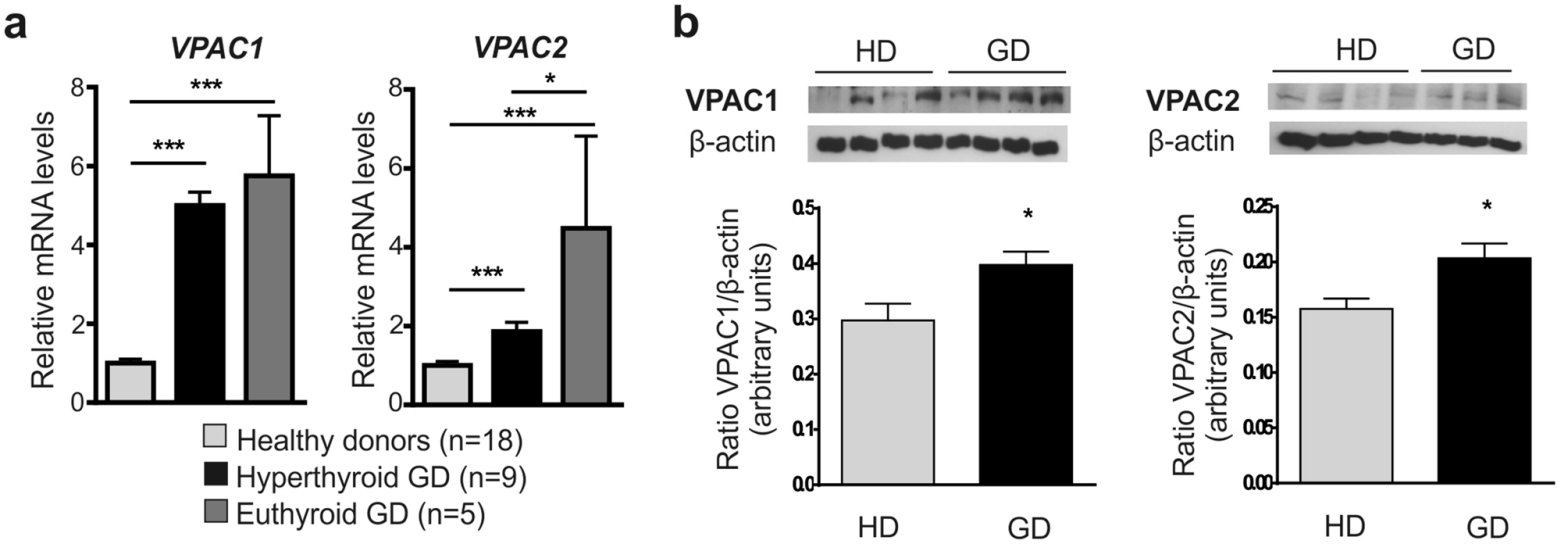
Characterization of VIP receptors in PBMC from healthy donors and from both hyperthyroid and euthyroid GD patients. (**a**) *VPAC1* and *VPAC2* mRNA expression levels in PBMC were determined by real-time PCR. Results are expressed as relative mRNA expression (relative to *GAPDH* mRNA levels) and referred to the expression level in healthy donors. Determinations were done in triplicate, and means and SEM are shown. (**b**) Protein levels of VPAC1 and VPAC2 in lysates of PBMC were measured by Western blotting. Protein bands were analyzed by densitometric analysis and normalized against the intensity of β-actin. Results are the mean ± SEM of at least 3 experiments and representative pictures are shown. *P < 0.05; **P < 0.01; ***P < 0.001.

We found that VPAC1 and VPAC2 transcripts levels were significantly upregulated in GD patients, in both hyperthyroid and euthyroid status when compared with healthy donors (Fig. 2a). Gene expression of VPAC1 was similarly high in both GD groups, whereas increase for VPAC2 mRNA expression was more pronounced in patients with normal thyroid status.

We then assessed protein expression of VIP receptors by means of Western blot. Results showed that VPAC1 and VPAC2 protein levels were significantly higher in PBMC from GD patients compared to healthy donors (Fig. 2b), in agreement with mRNA results. We then explored if these changes in receptor expression entailed an alteration in their function. As AC signalling pathway is the major transduction route mediating VIP effects, intracellular cAMP accumulation in PBMC was measured following stimulation with VIP or VPAC selective agonists (Table 2). We found that cAMP production elicited by VIP was significantly reduced in PBMC from hyperthyroid GD patients compared with healthy donors, whereas PBMC from euthyroid GD patients displayed a non-significant decrease in the VIP potency to stimulate AC activity. VPAC1 agonist-induction of intracellular cAMP was significantly decreased in all GD patients, being almost abolished in the euthyroid group. Conversely, VPAC2 agonist maintained its functional capacity to induce cAMP accumulation and showed an improved ability in euthyroid patients, reaching levels comparable to those induced by VIP in PBMC from healthy donors. Collectively, our findings showed an increased expression of VPAC1 and VPAC2 receptors in PBMC from GD patients. Hyperthyroid GD patients exhibited an impairment in VIP signalling not found in the euthyroid group which displayed a higher expression and improved functionality of VPAC2 receptors.

**Table 2:** Effect of VIP, VPAC1 agonist, and VPAC2 agonist on intracellular cAMP levels in PBMC. Intracellular cAMP levels were determined by ELISA in PBMC unstimulated or stimulated for 60 min with VIP (10 nM), VPAC1 agonist (10 nM), or VPAC2 agonist (10 nM). Determinations were done in duplicate.

	Healthy donors (n = 18)	Hyperthyroid GD (n = 9)	Euthyroid GD (n = 5)
Basal	0.38 ± 0.09	0.24 ± 0.05	0.31 ± 0.23
VIP, 10 nM	1.34 ± 0.22	0.42 ± 0.85*	0.51 ± 0.09
VPAC1 agonist, 10 nM	1.76 ± 0.25	0.47 ± 0.05**	0.05 ± 0.04***
VPAC2 agonist, 10 nM	0.97 ± 0.18	0.97 ± 0.23	1.6 ± 0.41

Data are expressed as pmol cAMP/mg protein, and means and SEM are shown. *P* values less than 0.05 were considered significant (*P < 0.05; **P < 0.001; ***P < 0.001), versus healthy donors. For more details, see “Methods” section.

### Thyroid hormone receptors are increased in PBMC from GD patients and its expression is related to the VIP system

Given that PBMC from both hyperthyroid and euthyroid GD patients exhibited a variation in VIP receptors, we next assessed whether thyroid hormone receptors were also altered. To that end, we examined the gene expression of the nuclear thyroid hormone receptors TRα and TRβ (*THRA* and *THRB*, respectively), and the plasma membrane integrin αvβ3 (codified by *ITGAV* and *ITGB3* genes) were examined in PBMC from patients with GD and healthy donors. Then, we studied its relationship with the VIP axis.

**Figure 3:**
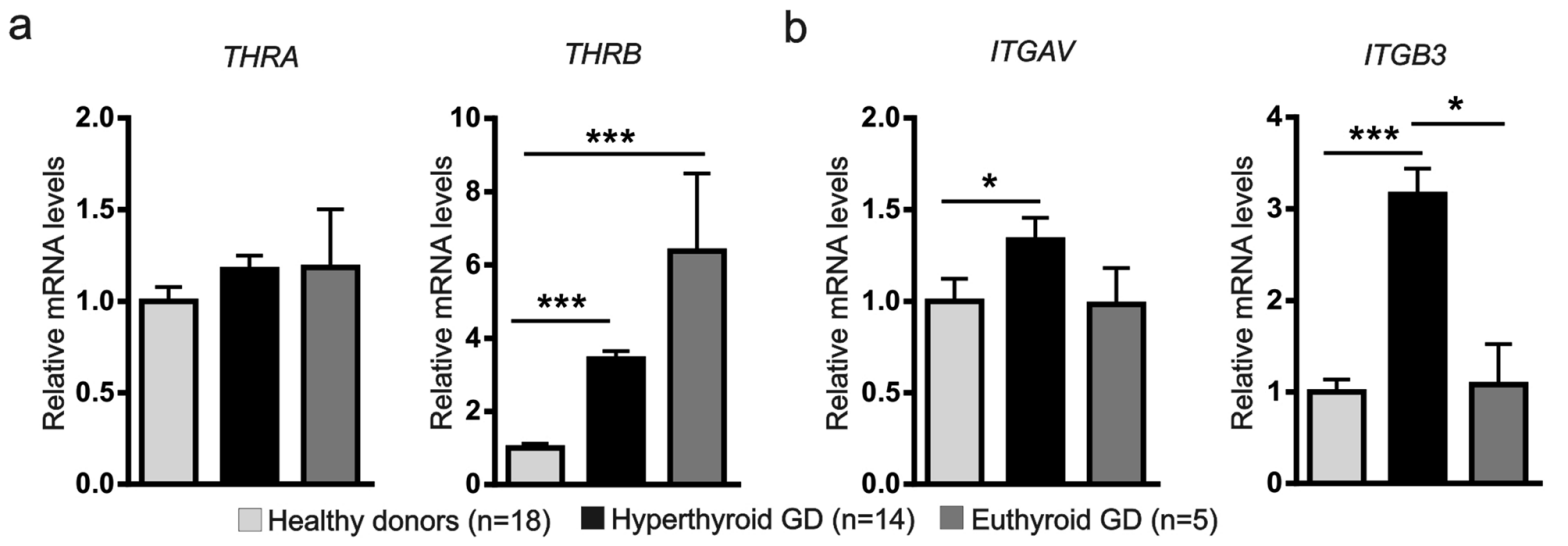
Gene expression of nuclear thyroid hormone receptors, TRα and TRβ, and plasma membrane integrin αvβ3 in PBMC from healthy donors and from both hyperthyroid and euthyroid GD patients. The mRNA expression levels of (**a**) *THRA* and *THRB*, and (**b**) *ITGAV* and *ITGB3* in PBMC were determined by real-time PCR. Results are expressed as relative mRNA expression (relative to *GAPDH* mRNA levels) and referred to the expression level in healthy donors. Determinations were done in triplicate, and means and SEM are shown. *P < 0.05; ***P < 0.001.

Transcript levels of thyroid hormone receptors, with the exception of *THRA,* were significantly increased in hyperthyroid GD patients compared to healthy donors (Fig. 3a, b).

When we explored the possible relationship between the altered expression patterns of receptors for VIP and for thyroid hormone, a significant correlation was found between the gene expression of *VPAC1* and all genes encoding thyroid hormone receptors in PBMC from healthy donors (Table 3). Specifically, there were positive correlations between mRNA expression levels of *VPAC1*, the nuclear receptors *THRA*, *THRB* and the *ITGAV* subunit of integrin, whereas a negative correlation was found with the subunit *ITGB3*. Conversely, none of these correlations were found in hyperthyroid GD patients, whereas euthyroid GD patients retained positive correlations with *THRA* and *ITGAV* while recovered the negativity in the correlation with *ITGB3* subunit, although not statistically significant (Table 3). Regarding *VPAC2*, only a significant positive correlation for *ITGAV* was found in healthy subjects, which was not observed in GD (Table 3). Hence, our results showed an imbalance in the expression pattern of VIP receptors and thyroid hormone receptors in PBMC from GD patients. The relationship observed between gene expression profiles of both types of receptors in healthy subjects was lost in the hyperthyroid group, whereas was partially retained in euthyroid patients.

**Table 3:** Correlation between gene expression of the receptors for VIP and for thyroid hormone in PBMC. Spearman’s correlation test performed with the relative mRNA expression levels of VPAC1 or VPAC2 and Thyroid hormone receptors.

	Healthy donors (n = 18)		Hyperthyroid GD (n = 9)		Euthyroid GD (n = 5)	
	*VPAC1*	*VPAC2*	*VPAC1*	*VPAC2*	*VPAC1*	*VPAC2*
*THRA*	0.3609**	− 0.0329	0.3059	0.4011*	0.8464***	0.2714
*THRB*	0.4597***	0.4152	0.1068	0.232	− 0.3107	− 0.0964
*ITGAV*	0.4911***	0.6923***	0.1148	0.3553	0.6321*	0.0678
*ITGB3*	− 0.4638***	− 0.2129	0.2637	0.243	− 0.0514	− 0.1429

Spearman’s correlation coefficients (rho) are shown, and statistical signification is marked as *P < 0.05; **P < 0.001; ***P < 0.001. For more details, see “Methods” section.

## Discussion

A complex network of immune-neuroendocrine regulatory interactions, mediated by hormones, neuropeptides and other signalling molecules, is crucial in the maintenance of homeostasis^1^. In this regard, potent anti-inflammatory and immunomodulatory effects of VIP have been demonstrated in several inflammatory/autoimmune diseases, where reduced serum levels of this neuropeptide and alterations in its signalling pathway in immune cells have been described^10,11,20^. Thyroid hormone levels, on their part, exhibit a positive correlation with markers of inflammation and immune activation, and thus, they may exacerbate the alterations of VIP axis^21^. However, the potential involvement of VIP in the particular case of autoimmune thyroid disease, which represent a model of autoimmunity and inflammation with abnormal thyroid hormones levels^22^ is not fully understood.

In the present study, we describe that VIP serum levels are significantly reduced in GD patients at the onset of the disease, when thyroid hormone levels are elevated (hyperthyroid status). On the contrary, no significant variations of VIP levels were observed in GD patients with normal thyroid status (euthyroid patients) or iatrogenic hypothyroidism. Levels of VIP were normalized in euthyroid GD patients independently of the treatment used to control hyperthyroidism, including antithyroid drugs, radioiodine or surgery. Accordingly, we also observed a negative correlation between VIP and FT4 serum levels in GD patients, whereas no significant relationships were found with other clinical parameters, including TSH, TgAb, TPOAb, and TRAb. It is worthy to note that variations in these clinical parameters are considered not specific for GD since can also be found in other AITDs and even in healthy subjects. However, thyroid hormone status of GD patients is evaluated on the basis of serum FT4 levels. Therefore, hyperthyroid GD patients show FT4 levels above the normal reference range whereas the euthyroid group has normal or near-normal hormonal values. In other words, our results convey that patients with an ongoing autoimmune thyroid hyperactivity exhibit significantly lower serum levels of VIP, suggesting that the increased serum FT4 levels could be a contributing factor to the decreased VIP levels in hyperthyroid GD patients. Our hypothesis is in agreement with previous reports in murine models which demonstrated modulatory effects of thyroid hormone on VIP mRNA levels in different adult brain areas^23^ and an upregulation of VIP content in the anterior pituitary gland under hypothyroid conditions^24–26^. Besides, our results link GD to other inflammatory/autoimmune diseases where VIP serum levels are decreased, such as juvenile idiopathic arthritis (JIA)^27^^,^ early arthritis^15^^,^ SpA^17^^,^ osteoarthritis^28^ and asthma^29^. Moreover, the fact that only the hyperthyroid subgroup with recent onset GD showed reduced VIP levels, suggests an association between low VIP levels and disease activity, as it has also been previously demonstrated in patients with early arthritis^15,16^, SpA^17^^,^ Chagas cardiomyopathy^30^ and JIA^27^. In this regard, GD activity has been associated with high levels of pathogenic Th17 and impaired Treg response^31–33^. These data would indeed be in accordance with the low VIP levels found at the onset of GD, given that VIP is able to promote Treg responses in several autoimmune diseases^34,35^, and induces a non-pathogenic phenotype in in vitro differentiated Th17 cells^36^. Therefore, it is worth speculating that GD might represent an additional autoimmune disease in which a disruption of VIP-mediated immune homeostasis could be linked to clinical outcome. Nevertheless, longitudinal studies in larger population samples would be useful to verify the potential role of VIP as a biomarker of GD activity.

Considering the hypothesis of an alteration of the VIP axis on immune cells, we characterized VIP receptors in PBMC from patients with GD and related these findings to thyroid status. Our findings showed an upregulation of VPAC1 and VPAC2 in GD patients as a group, in accordance with the dynamic regulation of both receptors reported in several autoimmune diseases, such as in PBMC and T cells from early arthritis patients^14,18^,in monocytes from rheumatoid arthritis (RA) patients^37^^,^in CD4^+^ T cells from multiple sclerosis (MS) patients^38^^,^ and in monocytes from Sjögren’s syndrome (SS) patients^39^. Despite the higher expression of VPAC receptors, VIP-stimulated signalling through VPAC1 receptor was significantly impaired in both hyperthyroid and euthyroid GD patients, whereas VPAC2 preserved its functional capacity. Interestingly, a remarkable increase in VPAC2 transcript levels was observed in PBMC from euthyroid GD patients compared to the hyperthyroid group. Therefore, the enhanced expression of VPAC2 could be considered as a mechanism that attempts to counterbalance the VPAC1 dysfunction by upregulating the expression of the functional receptor. Furthermore, VPAC2 agonist also displayed an improved ability to increase intracellular cAMP in euthyroid GD PBMC compared with healthy donors, suggesting a reinforcement of VPAC2 mediated signalling. This compensatory mechanism through VPAC2 would not be operating in the hyperthyroid group, which exhibited a impairment in VIP signalling. These data are in agreement with the role of VPAC2 in mediating VIP anti-inflammatory effects in synovial fibroblasts from RA patients that showed a reduced expression of VPAC1^40^. Moreover, our results point that, also in GD, VPAC2 expression is probably related to cellular activation and/or pathological conditions^19^. In this sense, VPAC2 also become the dominant receptor in PBMC, activated memory Th cells and Th17-polarized cells from early arthritis patients^14,18^. Moreover monocytes from SS patients exhibit higher expression of VPAC2, which is absent in healthy donors monocytes^39^^,^ and activated CD4^+^ T cells isolated from patients with MS also show a remarkable increase in the expression of VPAC2^38^.

Regarding thyroid hormone receptors, as far as we know, our results reveal for the first time an altered expression pattern of these receptors in PBMC from GD patients. Transcript levels of the nuclear TRβ receptor were significantly upregulated in all GD patients, whereas plasma membrane receptor αvβ3 was only increased in hyperthyroid GD patients. When we examined the interplay between the systems involving VIP and thyroid hormones in healthy subjects, we found a positive correlation between the relative expression of VPAC1 and thyroid hormone receptors mRNA, with the exception of the integrin β3 subunit which exhibited a negative correlation. Such negative relationship may reflect a limiting interaction between expression of *VPAC1* and *ITGB3*, by which the presence of the integrin heterodimer on the plasma membrane might be regulated. Interestingly, all correlations were lost in hyperthyroid GD patients, whereas the euthyroid group recovered the positive correlations with *THRA* and *ITGAV* genes, and also, the negativity in the correlation with *ITGB3*, although not statistically significant. Therefore, the restoration of such immune-neuroendocrine interactions in patients with normal thyroid status after medical treatment could be interpreted as an additional evidence of the direct relationship between these systems^1^^,^ suggesting the existence of reciprocal regulatory mechanism.

**Figure 4:**
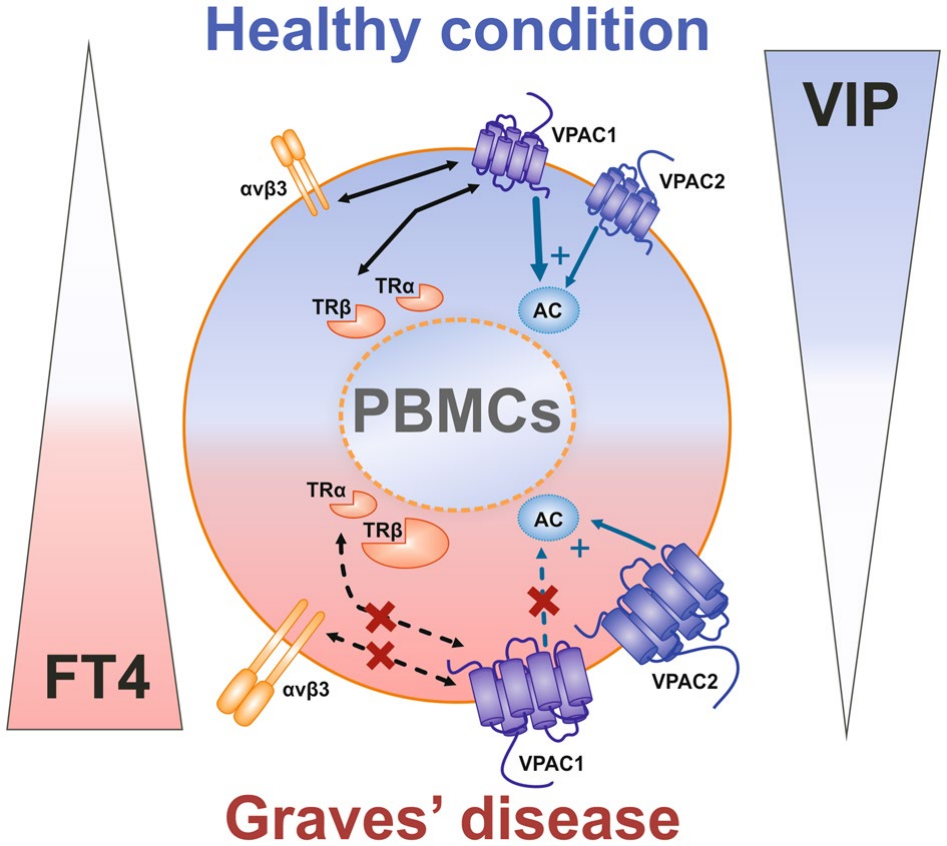
Graphical schematic representation for the proposed inter-relationship between thyroid hormone and VIP system in GD patients. VIP serum levels are significantly lower and negatively correlated with FT4 in hyperthyroid GD patients. Transcript levels of VIP receptors (VPAC1 and VPAC2) and thyroid hormone receptors (nuclear TRβ and plasma membrane αvβ3) are increased in GD patients compared to healthy donors, with the exception of TRα. GD patients show an impairment of VIP signalling through VPAC1 (dotted blue arrows), whereas VPAC2 maintains its capacity to stimulate AC activity (solid blue arrows). There is a correlation between the relative expression of VPAC1 and thyroid hormone receptors in healthy donors (solid black arrows) which is lost in hyperthyroid GD patients (dotted black arrows), suggesting the existence of interactions between both systems in this group of patients. (*PBMC* peripheral blood mononuclear cell, *FT4* free thyroxine 4, *AC* adenylate cyclase. Blue arrows indicate the contribution of each VPAC receptor signalling to AC activation. Bi-directional black arrows represent correlation between VPAC1 and thyroid hormone receptors).

In summary, our findings of reduced serum VIP levels and dysfunctional VPAC signalling in PBMC from hyperthyroid GD patients, suggest the existence of a dynamic connection between the neuroendocrine and immune systems and a prominent role of VIP in this setting (Fig. 4). Although further studies are needed to corroborate this hypothesis, our results represent an initial step to unravel the neuroendocrine-immune regulatory interactions in the specific setting of GD, which could certainly open new opportunities for therapeutic intervention.

## Methods

### Study population

We evaluated 222 patients (177 women, 79.7%) with AIDT: 78 with HT and 144 with GD. Diagnosis was established on commonly accepted clinical and laboratory criteria^41^. Specifically, diagnosis of HT was made when TSH levels were above the upper limit of normal (> 5 uU/ml), with or without low serum free T4 (FT4), and positive antibodies against thyroperoxidase (TPOAb > 100 U/ml) and/or thyroglobulin (TgAb > 344 U/ml). GD, on its part, was diagnosed when TSH levels were below the lower limit of normal (< 0.27 uU/ml), with or without elevated FT4 (> 1.7 ng/dl) and positive TSH Receptor antibodies (TRAb > 1U/ml). Another 49 healthy subjects, matched for age and sex, were included as controls. Complete clinical and demographic data were collected for all patients, including age, sex, history of tobacco, history of other autoimmune diseases, presence of thyroid diseases in other members of the family, goiter, orbital alterations, and previous therapies (anti-thyroid drugs, radioiodine, surgery or levothyroxine). No patient had been previously treated with steroids. 47 patients (21.2%) acknowledged a smoking habit and 60 patients (27.0%) recalled some sort of past family medical history regarding thyroid disease.

Clinical status at the time of blood sampling was evaluated in each of the 144 patients with GD and categorized as follows. We considered patients as “hyperthyroid GD, initial onset” when blood was collected at the time diagnosis and FT4 levels were above the normal reference limit (1.7 ng/ml); “euthyroid GD”, when patients were on anti-thyroid drugs and had normal or near-normal hormonal values; and “hypothyroid GD” for patients who developed hypothyroidism as a consequence of overtreatment with antithyroid drugs, or after therapy with radioactive iodine or surgery if they were not yet adequately replaced with thyroid hormone. Regarding treatments received by GD patients: 36 patients (25.0%) had received anti-thyroid drugs, 21 (14.6%) radioiodine and 14 (9.7%) had undergone surgery. The remaining 73 patients (50.7%) were naïve to any thyroid-directed treatment.

An informed consent was obtained from all patients participating in the study.

All the procedures were reviewed and approved by the Research Ethics Committee of Instituto de Investigación Sanitaria La Princesa (Madrid, Spain), and were in accordance with the 1964 Helsinki declaration and its later amendments or comparable ethical standards. All patients signed an informed consent form before sampling.

### Clinical, hormonal and autoantibody evaluation

Serum levels of FT4, TSH and TgAb, TPOAb, and TRAb were analyzed for each patient. FT4 levels were measured by radioimmunoassay (RIA) with Amerlex FT4 RIA kit (Trinity Biotech); plasma TSH was determined by a highly sensitive radioimmunometric assay Diagnost hTSH (Boehring Co); levels of TRAb were measured by enzyme-linked immunosorbent assay (ELISA) (DRG Instruments GmbH); levels of TgAb and TPOAb were assessed by the immunoradiometric assays ImmunoCAP Thyroglobulin and Immuno-CAP Thyroid Peroxidase kits (Phadia AB).

### Evaluation of levels of vasoactive intestinal peptide

We assessed levels of VIP using a commercially available competitive ELISA kit (Phoenix Pharmaceuticals), as previously described^17^. Serum samples were freeze-dried and dissolved in ELISA buffer (2:1). Levels of VIP were determined applying the corresponding dilution factor. Samples from each patient were assayed twice. The minimum detectable concentration was 0.12 ng/ml, with an intra-assay and inter-assay variation of ≤ 5 and 15%, respectively.

### Human peripheral blood mononuclear cells

PBMC from healthy donors, hyperthyroid GD patients and euthyroid GD patients were isolated from heparinized peripheral blood by density gradient centrifugation on Ficoll-Hypaque (Sigma Aldrich).

### RNA extraction, cDNA synthesis and real-time polymerase chain reaction (PCR)

As previously described in Carrion et al.^13^^,^ total RNA was extracted using TriReagent method (Sigma Aldrich). RNA quantity and purity were measured on a NanoDrop and 2 µg were used for cDNA synthesis using a High Capacity cDNA Reverse Transcription Kit (Life Technologies). Real-time PCR analysis for all target genes and one house keeping gene (*GADPH*) were performed using TaqMan Gene Expression Master Mix (Life Technologies), with manufacturer-predesigned primers. Assays were made in triplicate, and results were normalized according to the expression levels of *GADPH*. Results were obtained using the 2-ΔΔCt method for quantification.

### Western blot analysis of VPAC1 and VPAC2 receptors

As previously described in Carrion et al.^13^ PBMC proteins were extracted in ice-cold radioimmunoprecipitation assay buffer (50 mM Tris–HCl pH 7.5, 150 mM NaCl, 30 mM NaF, 5 mM EDTA, 1% Triton X-100, 1% NP-40, 0.1% SDS, 1 mM DTT, 1 mM sodium orthovanadate, protease inhibitor cocktail). 30 μg and 40 μg protein extracts, for VPAC1 and VPAC2 respectively, were subjected to 10% SDS-PAGE and transferred to PVDF membranes (BioRad). After blocking, membranes were incubated overnight at 4ºC with rabbit polyclonal anti-human VPAC1 (1: 15,000; Thermo Scientific) and mouse monoclonal anti-human VPAC2 (1: 1000; Abnova Corporation). Mouse anti β-actin (1:20,000, Sigma-Aldrich), was used as a loading control. Horseradish peroxidase conjugated secondary antibody (1:10,000, Santa Cruz Biotechnology) was used. Proteins were detected using Western Blotting Luminol reagent (Santa Cruz Biotechnology), analyzed using the Bio-Rad Quantity One program and normalized against β-actin.

### Determination of intracellular cAMP concentrations

Levels of cAMP were determined by an ELISA kit (ADI-900-066, Enzo Life Sciences). PBMC obtained from 18 healthy subjects, 9 hyperthyroid GD patients and 5 euthyroid GD patients were cultured in RPMI-1640-GlutaMAX media (Life Technologies) supplemented with 10% FBS (Lonza,) plus 1% penicillin/streptomycin. After cells were treated with 10 nM VIP (Polypeptide Group), VPAC1 agonist [Lys^15^Arg^16^Leu^27-^VIP (1-7)-GRF (8-27)], or VPAC2 agonist (RO 25-1553) (Bachem) for 60 min, the reaction was terminated by aspiration of the growth medium and addition of 0.1 N HCl. The concentrations of cAMP in the cell lysates were measured according to the manufacturer’s instructions. Protein concentration was determined using a QuantiPro BCA Assay Kit (Sigma- Aldrich).

### Statistical analysis

Descriptive results are given as mean ± standard deviation and median (interquartile range) for normally- and not-normally-distributed continuous variables, respectively. Categorical variables were summarized as frequencies and percentages.

In order to overcome the *batch effect*, which is intrinsic to ELISA determinations, the results obtained for VIP levels were homogenized. Association of VIP levels with patients’ characteristics was assessed using two-sided analysis of variance (Kruskal–Wallis or *U*-Mann Whitney tests, as required), general linear models, logistic and linear regression analysis and bivariate correlations (Spearman).

All statistical analyses were performed using SPSS version 21.0 (IBM SPSS Statistics Inc.). The *P* values were two-sided and statistical significance was considered when *P* < 0.05. Significance of results was analysed using the GraphPad Prism software version 6 (Graphpad Software Inc.). Data were subjected to normality test (Kolmogórov-Smirnov test) and equal variance test (*F*-test). Mann Whitney or Kruskal–Wallis test was used in intergroup comparison between not-normally-distributed continuous variables. *P* values less than 0.05 were considered significant (*P < 0.05; **P < 0.01; ***P < 0.001). Results are expressed as the mean ± standard error of the mean (SEM).

## Data Availability

All relevant data are within the paper. The datasets generated and/or analysed during the current study are not publicly available due to the confidential nature of the clinical data but are available from the corresponding author on reasonable request.
